# Australian Livestock Export Industry Workers’ Attitudes toward Animal Welfare

**DOI:** 10.3390/ani11051411

**Published:** 2021-05-14

**Authors:** Renee S. Willis, Emma J. Dunston-Clarke, Leah R. Keating, Patricia A. Fleming, Teresa Collins

**Affiliations:** 1 Food Futures Institute, Murdoch University, Murdoch, WA 6150, Australia; Emma.Dunston@murdoch.edu.au (E.J.D.-C.); leah_257@outlook.com (L.R.K.); t.collins@murdoch.edu.au (T.C.); 2 Harry Butler Institute, Murdoch University, Murdoch, WA 6150, Australia; t.fleming@murdoch.edu.au

**Keywords:** stockperson, stock handler, husbandry, transportation, societal perceptions, farm animal welfare

## Abstract

**Simple Summary:**

Societal concern for the welfare of animals in the Australian live export industry is substantial, with particular concern shown for animals after they have left Australian shores. The competency of stock handlers is recognized as one of the most important factors affecting the welfare of exported livestock. Therefore, a survey of 265 industry workers was undertaken to gauge their understanding of animal welfare, their attitudes toward welfare in their workplace, and to identify any differences according to their industry role. Surveys were disseminated in six languages to twenty different supply chain locations in Australia, Southeast Asia, and the Middle East. The majority of respondents showed a strong understanding and positive attitude toward animal welfare concepts. Participants generally felt that livestock welfare was important to them and that livestock should be treated with respect. Responses were analyzed according to participants’ role in industry, finding that there was minimal variation in beliefs or attitudes between supply chain roles, and no specific group was found to differ from others consistently. The majority of participants identified ways they had positively impacted livestock welfare in the past and provided suggestions for additional improvements within industry. These findings may be useful for addressing the discordance between societal perceptions and those of industry workers.

**Abstract:**

Understanding live export industry workers’ attitudes and beliefs toward animal welfare can provide insight into their decision-making processes and likely behavior. Industry workers (*n* = 265) with various roles within the supply chain were surveyed from different global regions. Participants were divided into ten categories according to their industry roles and compared using ordinal regression. Respondents were highly likely to have a positive attitude toward animal welfare; the majority of workers enjoyed working with livestock (95.8%) and agreed that livestock should be treated with respect (97.7%). Workers demonstrated a strong understanding of animal welfare concepts, 168 respondents (63.4%) provided examples of ways they had improved animal welfare in their workplace, and 164 workers (61.9%) suggested ways that animal welfare could be improved further. Most workers (95.8%) agreed that animal welfare was satisfactory in their workplace. Five out of the 24 multiple-choice responses differed significantly by the participant’s industry role, but no particular group displayed consistently divergent beliefs or attitudes. Given the community concern regarding animals in the livestock export supply chain, it is imperative to understand the attitudes of industry personnel who are responsible for the daily management of the animals. This knowledge assists in the development of animal welfare policy and can inform strategies to manage public perception.

## 1. Introduction

The welfare of livestock exported by sea is of particular interest to the Australian public. The live export industry receives frequent attention in the media and from animal advocacy groups [1,2]. Significant concern has been generated by media attention highlighting incidents of poor handling and inhumane slaughter practices in countries receiving Australian livestock [1,3,4]. Human–animal interactions are recognized as a significant contributor to welfare outcomes during livestock transport and in production animal systems [5,6]. The interactions between livestock and their handlers can positively or negatively affect animal well-being [7]. In the livestock export industry, workers implement procedures and regulations regarding animal health and welfare and make critical decisions that directly impact livestock throughout the supply chain (e.g., daily monitoring of livestock, provision of resources and health care, reporting on health and welfare outcomes as stipulated under industry regulatory requirements). Previously, members of the general public, animal welfare advocates, and those working in the industry have ranked the attitude and competency of stockpersons as two of the top three most important industry welfare factors [8]. Livestock export involves transporting animals to multiple nations globally. Consequently, exported animals move between several supply chain facilities and are regularly handled and managed by workers from different cultural backgrounds with a range of stock handling expertise. Furthermore, human–animal interactions and management practices early in the export supply chain can exacerbate welfare outcomes as animals are exposed to intense handling and different management conditions when moving between facilities along their journey [9].

Animal handling practices have previously been identified as a crucial factor influencing animal welfare in the live export industry [10,11]. In 2011, the Export Supply Chain Assurance System (ESCAS) was introduced in response to poor handling and slaughter practices in many facilities receiving Australian livestock [11,12]. ESCAS aims to ensure that animal handling and slaughter practices for Australian livestock in importing countries meet the World Organization for Animal Health (OIE) recommendations. Despite the introduction of ESCAS, surveys of the public have identified ongoing concern for the handling of animals after leaving Australian shores [8,13]. The live export industry is aware that public perception and evolving community values regarding animal welfare need to be addressed [3,14]. Surveys of public attitudes toward the livestock export industry demonstrate that some community members believe industry workers do not have high regard for animal welfare [13]. This follows a broader trend, whereby the role of stock handlers in production animal systems is often undervalued [15]. Surveys have been undertaken to assess society’s opinions on animal welfare in the livestock export industry [14,16,17]; however, studies to investigate industry workers’ attitudes toward livestock welfare and how attitudes may differ between supply chain sectors are limited [8,18]. Despite the importance of their role(s), industry workers are often overlooked during decision making processes or regulatory reform. However, workers can be a constructive resource for identifying welfare issues and implementing possible solutions [15,19]. Stock handlers’ attitudes, beliefs, and comprehension of animal welfare are integral drivers for determining the likely nature of their interactions with livestock [15,20].

A person’s behavior is influenced by their own beliefs and attitudes, by the values of those around them, and their perceived ability to act in a particular manner [21,22]. It is well established that these principles apply to stock handlers [5,21,23]; therefore, analyzing these elements can indicate the expected nature of stock handlers’ interactions with livestock, and their impact on animal welfare. Previous studies have also identified factors that influence the attitudes of stock handlers toward animal welfare, and these may apply to live export workers. Southeast and East Asian stakeholders identified demographic factors such as gender, religion, and previous experience with farm animals as influential [24,25,26]. In addition, level of training [5,26,27], working conditions such as salary, respect, and job satisfaction, can impact worker attitudes [15]. Therefore, understanding worker attitudes and beliefs at different supply chain sectors may help identify factors that can be modified or improved.

This study surveyed participants who worked at various points in the Australian livestock export supply chain aiming to: (1) examine workers’ attitudes and understanding of animal welfare; (2) determine their perception of animal welfare in their workplace; and (3) ascertain whether workers’ attitudes were influenced by their role in the supply chain.

## 2. Materials and Methods

### 2.1. Participant Recruitment and Survey Distribution

This study was approved by the Murdoch University Human Research Ethics Committee (2018/022). Researchers used an information sheet and verbal communication to confirm the aims, informed consent, voluntary nature, and individual confidentiality of the collected information. All survey questions were optional.

**Figure 1:**
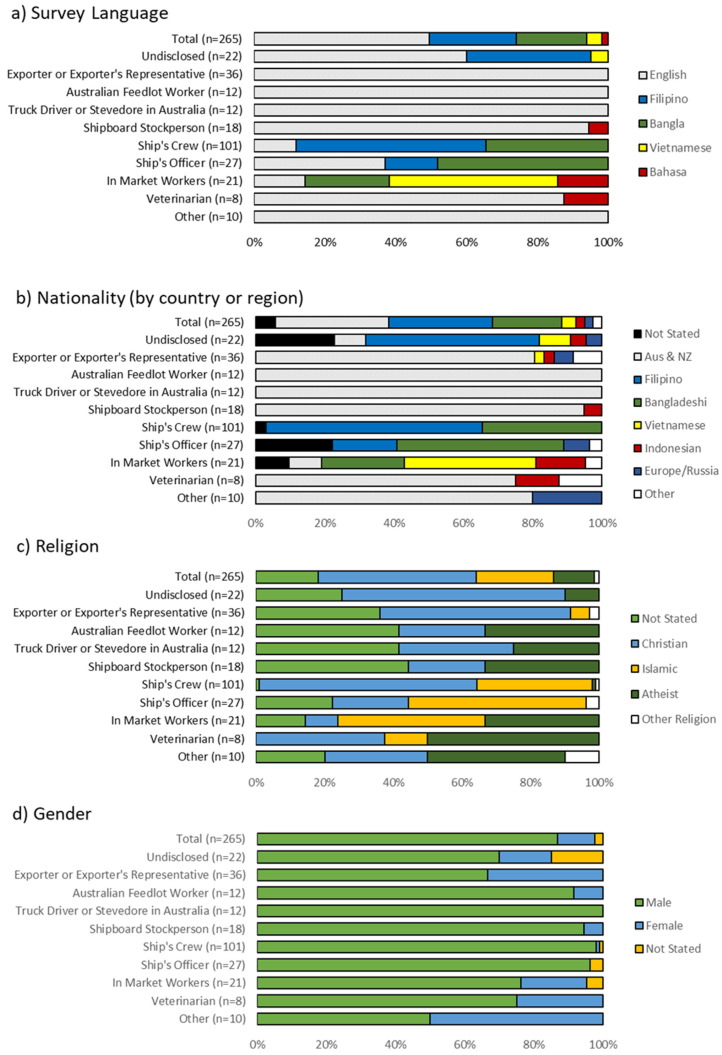

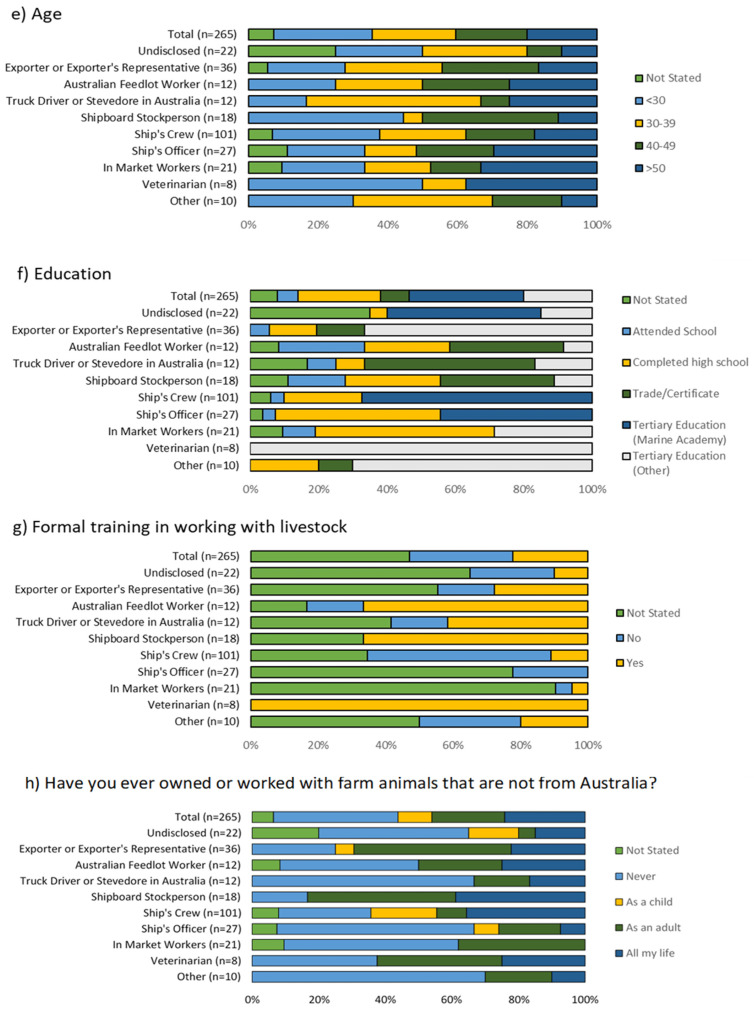
(**a**–**h**) Demographics by role in industry.

Participants were invited and eligible for recruitment if they were employees in the Australian livestock export industry supply chain, either in Australia and overseas. The survey design was based on other published surveys testing livestock handlers’ attitudes to animals [23,25,28,29] and incorporating the Theory of Planned Behavior [22,30]. Questions were adapted to suit the livestock export context and divided into three parts:

- Part 1: Ten demographic questions (e.g., age, nationality, role in industry) (Figure 1).
- Part 2: Eighteen questions on attitudes and personal beliefs toward animal welfare and animal sentience [28]; livestock handling and acceptable workplace practices [23]; and euthanasia of livestock [25] (Table 1).
- Part 3: Six questions regarding the respondent’s opinions on their current working environment and the welfare of livestock within it [29], as well as their perception of co-workers (Table 1). In addition, two short answer questions were asked on how the welfare of exported animals could be improved and ways that the participant had improved animal welfare in the past (if any).

Survey responses were measured on a five-point Likert scale from ‘strongly agree’ to ‘strongly disagree’ for questions regarding opinions on welfare, and ‘all of the time’ to and ‘never’ for questions on handling livestock. Questions in Parts 2 and 3 were presented to reduce bias by randomly alternating between affirmative and negative answers and between questions on both cattle and sheep; this enabled identification of whether participants had interpreted the questions consistently. Respondents were also asked to provide examples of their experiences and welfare considerations in a free text format. Providing meaningful short answer responses required an understanding of animal welfare issues and reduced the participant’s ability to choose proposed answers based on what was perceived as socially acceptable [26].

The survey was translated to Arabic, Bangla, Vietnamese, Filipino, and Bahasa Indonesian languages, with respondents able to select their language to ensure the accuracy of understanding. Surveys included a definition for ‘euthanasia’ and ‘welfare’ to facilitate respondents’ interpretation of questions.

Flyers advertising the survey were distributed to workplaces such as feedlots in Australia and overseas, at Fremantle Port during the loading of livestock carrier vessels, and on-board during voyages. Convenience sampling was also employed, with data collection dependent on scheduled livestock consignments from Australia during the data collection period (August 2018 to April 2019). Surveys were distributed by email link from the chief investigator to export company managers, and in paper form by four Australian Government Accredited Veterinarians, one Australian Accredited Stockperson and two industry researchers. To maintain confidentiality and encourage participants to respond honestly, surveys were completed anonymously, with hard copy surveys enabling workers to answer at their own pace and return surveys in a closed folder or envelope.

Permission to distribute surveys and flyers was sought from export company directors, ship captains, or feedlot managers. Data were collected from participants working at three major exporting companies, on ten ships, six feedlots (four Australian and two foreign feedlots), and an industry (LiveCorp) stockpersons’ accreditation course in Fremantle, Western Australia. Respondents included exporters and their agents, stock handlers working in feedlots, on ships and at the wharf, road transport drivers, veterinarians, importers and their agents, producers, suppliers, and those in administrative roles. One export company declined the invitation to participate in the survey.

To determine the minimum required sample size, we generously estimated a sample population of 8000 industry workers whose roles involved livestock welfare within the Australian live export industry supply chain. We then applied a confidence interval of 95% and a margin of error at less than 10% (7%) and calculated192 participants as the minimum number of survey participants required [31]. Not all respondents answered all questions; however, the minimum number of participants was easily met in each case.

### 2.2. Statistical Analysis

Survey responses were combined into an Excel spreadsheet [32] and checked for robustness with question comprehension and inconsistent answers. Descriptive statistics were generated by numerically coding data responses. Responses for each Likert scale score were reported for all questions (Table 1). The percentages reported in the text were calculated by pooling Likert scale answers in agreeance (Scores 1 and 2), reporting the percentage of undecided respondents, or by calculating the percentage of responses disagreeing with each question (Scores 3 and 4).

‘Industry role’ described the sector within the supply chain where a worker was engaged. Participants were divided into ten categories according to their industry roles and grouped as: exporters or exporter’s representatives; Australian feedlot workers; truck drivers or stevedores in Australia; shipboard stockpersons; ship’s crew; ship’s officers; workers ‘in-market’ (at feedlots or companies in countries receiving Australian livestock); veterinarians; those who did not disclose their role; and ‘other’, which were those that reported positions that did not fit into the categories provided (such as administrative roles, suppliers to the industry, or producers). Many participants gave multiple responses to the question *What is your role in industry?* (*n* = 308); therefore, responses were ordered so that their role was listed under the category with the fewest survey respondents. Statistical analysis via ordinal regression using SPSS, version 24 [33] was undertaken on Likert scale responses (Parts 2 and 3) with industry role entered as a categorical covariate and responses entered as the dependent variable.

Responses to open-ended questions were organized into categories based on themes identified within the comments. Responses regarding the welfare improvements identified were categorized as provision of feed and water; provision of good housing; improved livestock management practices; training and education; ensuring livestock health; improving company policies; and preparation of livestock for transport. Suggestions for improving the welfare of exported animals were grouped as adequate provision of feed and water; improvements in housing; facilities and infrastructure; and better preparation and selection of livestock. These responses were analyzed by comparing the number of comments identified under each category.

## 3. Results

### 3.1. Demographics of Respondents

Of the 265 responses received, many were completed in English (49%), with the remainder in Filipino (25%), Bangla (20%), Vietnamese (4%), and Bahasa (2%) (Figure 1a). The predominant nationalities were Filipino (29.8%), Australian and New Zealander (32.8%) and Bangladeshi (20.0%) (Figure 1b). Filipino respondents either worked on ships (comprising 62.4% of the crew demographic and 18.5% of the ship’s officers) or did not disclose their industry role. Australian and New Zealand participants worked in all industry roles except for the ship’s crew or officers. The Bangladeshi workers contributed to 34.6% of the ship’s crew and 51.9% of the ship’s officers. The remaining Bangladeshi participants (13.5%) worked in-market. The Filipino respondents predominantly identified as Christian (97.5%) and Bangladeshi respondents as Muslim (98.1%). Australian respondents were predominantly Christian (44.0%) or atheist (25.3%), while 28.0% choose not to respond to this question (Figure 1c).

Participants were mostly male (86.8%); exporters or their representatives and the category ‘other roles’ had the highest representation of women (33.3% of 36 respondents, and 50% of 10 respondents, respectively) (Figure 1d). There was a relatively even distribution of ages, from below 30 years of age (28.3%), 30–39 years (24.2%), 40–49 years (20.4%) and more than 50 years (20.0%) (Figure 1e).

Roles identified were exporters or their representative (13.6%); Australian pre-export feedlot workers (4.5%); truck drivers or stevedores (4.5%); ship’s crew (38.1%); officers (10.2%) and shipboard stockpersons on-board livestock vessels (6.8%). Other roles were in-market workers at feedlots or companies that received Australian livestock (7.9%), veterinarians working across all areas of the supply chain (3.0%), while 3.8% of respondents worked in other roles such as administration or finance. A proportion of respondents (7.6%) chose not to disclose their role in industry.

Most respondents had either completed high school (24.2%) or had higher education in the form of a trade certificate or tertiary degree (61.9%) (Figure 1f). Despite high levels of further education or training, very few respondents declared that they had received any formal training with livestock (22.3%) (Figure 1g). Besides veterinarians and shipboard stockpersons (whose roles require formal training), Australian feedlot workers were most likely to report formal training (66.6%). In comparison, 10.9% of ship’s crew and none of the ship’s officers reported having any formal training with livestock. This question had the highest non-response rate for the survey (*n* = 125).

Although many workers did not have formal training with livestock, they had extensive experience with farm animals, either in Australia or other parts of the world. Of the 67.2% of respondents who were not Australian or New Zealander by nationality (*n* = 178), 53.9% reported that they had experience working with farm animals not from Australia (Figure 1h).

### 3.2. Overall Attitudes to Welfare

When answering questions on beliefs about animal welfare, participants in all sectors showed a high level of agreement when responding to the statements *working with livestock is enjoyable to me* (95.8%), *the welfare of livestock in my workplace is important to me* (98.9%), and *livestock should be treated with respect* (97.7%). When considering sentience, only 5.0% of participants disagreed with the statement *livestock have feelings,* and 21.3% of participants agreed with the statement *livestock do not form relationships with humans.* Only 0.8% disagreed with the statement *I can see if livestock are stressed.* Participants’ understanding of pain perception in livestock was reflected by only 8.1% agreeing with the statement *cattle don’t feel pain*, and only 2.3% agreeing with the statement *sheep don’t feel pain*. When considering euthanasia, 80.2% of participants agreed that *euthanasia was sometimes necessary in cases of sick or injured livestock*, while 10.1% were undecided, and 9.7% disagreed.

Statements concerning knowledge around animal handling and management showed most participants demonstrated a good understanding of the needs of animals, and replies were consistent with favorable livestock handling practices. Attitudes toward acceptable animal handling were reflected by responses to the statements: *hitting cattle helps when moving them* (only 2.3% agreed); and *it is acceptable to pull a sheep by a leg to move them* (2.4% agreed) (Table 1). Most (90.3%) participants agreed that *it is important to move livestock* slowly, 93.5% agreed that *it is important for livestock to be able to lie down to rest*, and 89.3% agreed that *when moving livestock, it is better to remain calm than shout in a loud voice* (Table 1).

### 3.3. Animal Welfare within the Workplace

When asked if *the welfare of animals is satisfactory in my workplace*, 95.8% of respondents agreed, 2.7% were undecided, and 1.5% disagreed (Table 1). Two-thirds of respondents (164 respondents or 61.9%) provided 256 ideas and suggestions when asked to outline ways to improve the welfare of exported animals. Workers gave responses regarding adequate provision of feed and water (59 comments), improvements in housing, facilities, and infrastructure (51 comments). Better preparation and selection of livestock was suggested in 39 comments. Examples of responses include: “*Increase feed onboard, especially chaff*”, “*Improvement or abolishment of older ships*,” and “*Cattle should be brought to the vessel in good condition. Mostly cattle that are brought onboard are very frightened of human beings*.” Five respondents stated that animal welfare in the livestock export industry is currently acceptable and did not have any suggestion for further improvement.

The respondents’ perception of co-workers and their consideration of acceptable workplace practices showed that 96.9% agreed that the *people in* [their] *workplace work hard to care for the livestock*. Assessment of perceived behavioral control and the ability to act in accordance with the beliefs highlighted above was reflected by agreement with the statement *I feel confident working with livestock* (88.4% agreed), and disagreement with the statement *livestock are uncontrollable* (90.0% disagreed). More respondents had positive rather than negative views toward their ability to effect change, with 66.7% disagreeing with the statement: *sometimes, I cannot do anything to improve poor animal welfare in my workplace* (Table 1).

Participants agreed (84.5%) that they had tried to improve the welfare of animals in their workplace in the past, while 10.0% were undecided (Table 1). Comments were given by 168 participants (63.4%) in response to the question: *Can you give an example of how you have improved animal welfare in your workplace*? Responses included 251 relevant examples of animal welfare improvements they had made in the past. Comments concerning the provision of feed and water were the most frequent (69), comments regarding provision of good housing or good livestock management practices were frequently given (54 comments on each), as were those on training and education (37). Further comments were made on ensuring livestock health, improving company policies, and preparation of livestock for transport. Examples include: “*Work place training to better my skills in livestock handling*” and “*We make sure we give them enough food and water regularly and clean their pens*.”

### 3.4. Understanding of Welfare by Role in Industry

**Figure 2:**
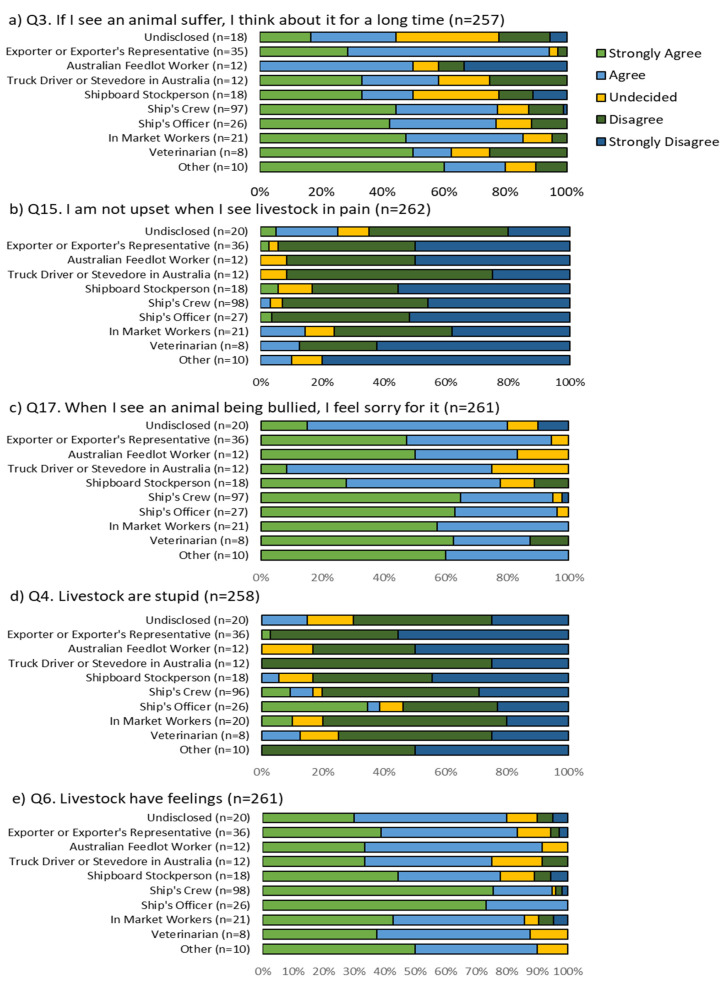
(**a**–**e**) Survey responses for questions that differed significantly by role in industry.

Five of the 24 Likert scale questions showed a significant difference between respondents’ workplace roles (Table 1). Of these, three were related to feeling empathy toward livestock in cases of poor welfare. Responses to the statement: *If I see an animal suffer, I think about it for a long time,* showed that although 50.0% of Australian feedlot workers agreed, unlike all other groups, no feedlot workers strongly agreed with the statement. Australian feedlot workers were also more likely to strongly disagree (33.3%) with this statement when compared to all other workplace roles (Figure 2a).

The majority of participants in all sectors disagreed with the statement: *I am not upset when I see livestock in pain.* However, participants who did not disclose their industry role showed more variability in their responses than other groups, with a wide range of scores (25% in agreement, 10% undecided, and 65.0% disagreed) (Figure 2b).

Few respondents from any group disagreed with the statement: *When I see an animal being bullied, I feel sorry for it*; however, there was a difference in the level of agreement between those in different industry roles. The majority of ship’s crew, ship’s officers, in-market workers, veterinarians, and those in ‘other’ positions strongly agreed with the statement. In contrast, truck drivers and stevedores in Australia, shipboard stockpersons, Australian feedlot workers, and the undisclosed group were more likely to select agree or undecided in response to the statement. Exporters and exporters’ representatives were evenly divided between agreed (47.2%) or strongly agree (47.2%) (Figure 2c).

The two remaining questions that showed a significant difference by workplace were related to beliefs about animal sentience. The majority of respondents in all groups disagreed with the statement: *livestock are stupid.* However, ship’s officers had a greater percentage of answers in agreeance (33.3% strongly agreed) than any other group (Figure 2d). Despite this finding, ship’s officers, along with ship’s crew, were more likely than other groups to strongly agree that *livestock have feelings* (74.4% and 73.2%, respectively) (Figure 2e). Furthermore, ship’s officers were the only group to agree with the statement unanimously.

Participants that disagreed with the statement *euthanasia is sometimes necessary in cases of injured or sick livestock* were ship’s crew (20 respondents), in-market workers (four respondents), or a ship’s officer (one respondent) (Table 1). However, as the majority of respondents in these groups agreed with the statement, there was no significant difference detected by industry role.

## 4. Discussion

The principal aim of the study was addressed by responses demonstrating that workers within the livestock export industry had a good understanding of welfare concepts and animal sentience and held positive attitudes toward the animals under their care. Participants also felt positive about their workplace and provided ways to improve welfare outcomes; there were no sectors with consistently convergent in their beliefs or attitudes, addressing our second and third aims, respectively.

Livestock export workers’ views on animal sentience were comparable to the 1521 members of the Australian public surveyed on farm animal welfare by Futureye [17]. The majority of the public agreed that animals have the capacity to experience stress (86%), have desires and wants (73%), and have the capacity to experience joy and pleasure (81%). Similarly, the livestock export workers in our survey agreed that they could see if livestock were stressed (92.7%) and that livestock had feelings (89.3%). Futureye [17] found that 87% of the Australian public believed that animals were aware of bodily sensations such as pain, heat, cold, or hunger. Similarly, the livestock export workers in our survey disagreed with the statements that cattle do not feel pain (91.1%), or sheep do not feel pain (96.1%), demonstrating their understanding of sentience. These comparable responses suggest that the views of livestock export industry workers captured in our study align with those of the Australian public.

Previous studies on human–animal relationships demonstrate that behavioral intent precedes displayed human behavior and is influenced by (1) having a positive attitude toward a behavior; (2) feeling that the behavior is a favorable social norm; and (3) having perceived behavioral control [21]. Responses to questions examining the participants’ attitudes and beliefs toward welfare concepts generally indicated positive attitudes and values, suggesting industry workers would endeavor to treat animals well. Questions assessing favorable social norms within the workplace highlighted that workers did not widely accept poor handling practices and commonly recognized that people worked hard to care for livestock. Workers also demonstrated that they had perceived behavioral control as they typically felt confident in their roles and expressed that they can take action to provide desirable welfare outcomes for the animals under their care. These findings show that a substantial majority of surveyed livestock export industry workers displayed positive intent toward taking measures to improve animal welfare.

It is not possible to verify how people will act in their workplaces based on survey data, and intention alone does not confirm how people will behave in a specific context. Sinclair et al. [24] surveyed stakeholders’ attitudes toward livestock transport and slaughter in Southeast and East Asia. Most participants in their study expressed positive attitudes toward improving animal welfare by indicating that welfare was important to them; however, fewer respondents agreed that they intended to make improvements to the welfare of animals. Similarly, Verrinder and Phillips [34] reported that although the veterinary students they surveyed felt strongly motivated to act in the interest of animals, many felt they had taken little affirmative action to improve animal welfare. The majority of workers completing this survey indicated that welfare was important to them, and most reported having improved animal welfare in the past. When subsequently asked to give specific examples of having done so, most participants could provide one or more responses. These examples were focused on key welfare principles such as provision of resources “*feeding them every day*”; care for the environment “*ensure ventilation 24 h*”; good stockmanship “*low stress animal handling*”; and continued training and education of workers “*teaching new employees how to handle stock and identify stressed animals*”. This sample of livestock export industry workers showed positive behavioral intent toward affirmative action, combined with evidence of past behavior focused on prioritizing animal welfare improvement in the workplace.

Respondents predominantly enjoyed working with livestock and felt positive about welfare within their workplace. These findings complement studies of workers in other production animal sectors that found people tended to enjoy working with livestock on a daily basis [5]. Furthermore, a 2021 industry survey on live exports indicated that the majority of workers showed a high level of acceptance of the industry and felt the industry listened to and respected community opinions [18]. Although the industry survey did not directly canvass worker’s views on animal welfare, this is a particular point of issue for the live export, and it can be assumed that their views on animal welfare would have strongly influenced responses on industry acceptability. The majority of respondents to our survey provided suggestions for ongoing improvements to animal welfare in their workplace, indicating that workers recognized welfare concerns and are a valuable source of information to enhance animal welfare outcomes for the industry.

It is well recognized that those who work in animal production tend to view welfare within their workplace more favorably than members of the public [35]. European studies comparing farmers’ views with those of the public found that farmers tend to assess welfare based on optimal health and access to resources within their management systems. However, the public tended to judge welfare outcomes based on optimal health, access to resources, and the ability for animals to engage in natural behavior [35,36]. The discordance between livestock export industry workers’ and the general publics’ views on the welfare of exported livestock is likely based on their interpretation of the importance of different components of welfare. Furthermore, industry workers base their perceptions on their day-to-day experiences of routine trade practices rather than information from media articles and advocacy campaigns centering on extreme events of poor welfare [1,3].

The effect of role along the Australian export supply chain was investigated, noting that diverse demographic groups represented each role. Gender has previously been reported as an influencing factor when assessing attitudes to animal welfare [25,26,27]. The gender of survey respondents reflected the male-dominated industry with shipboard workers, truck drivers, and stevedores, and Australian feedlot workers being predominantly male; however, other roles were more gender diverse. Despite the different gender demographics between roles, no differences in survey responses were found. Roles varied in religiosity, with participants from Australia or New Zealand less likely to hold religious beliefs than those of other nationalities who were predominantly Christian or Islamic. Religion has been reported as an influencing factor for beliefs regarding transport and slaughter of livestock [24,25] but did not affect responses in our study. Formal training in livestock handling has been recognized to influence attitudes toward animal welfare [5,26,27]; however, despite the discrepancy in formal training reported by workers, this had limited influence on our results.

Despite the overall convergence of beliefs and attitudes across industry roles, there was some subtle variance. When considering animal sentience, all ship’s officers and most ship’s crew (95.0%) agreed or strongly agreed that livestock have feelings; however, ship’s officers were more likely than others to agree that livestock are stupid. These results may reflect ship officer’s interactions with livestock: although they frequently make decisions that impact animal welfare, officers often have limited experience directly handling livestock and observing the nuances of livestock behavior [37]. Notably, three of the five questions showing a significant effect of industry role were statements requiring self-reflection. These questions required an awareness of the animal perspective as well as the respondent’s own emotional reaction to a situation. Although workers in Australian feedlots, truck drivers, and stevedores in Australia, and those in undisclosed roles all expressed agreement with questions supporting animal sentience, when asked if they were personally affected by poor animal welfare, these respondents were less likely to agree. These findings may indicate differences in the way respondents perceive their own emotions rather than the animal welfare concepts presented in the question. Regardless of their role in the supply chain, respondents generally demonstrated a good understanding of animal welfare and indicated that welfare was an important concern to them. It is notable that some respondents did not agree that euthanasia was sometimes necessary in cases of injured or sick livestock. As this question was primarily answered favorably, there was no significant difference identified by industry role; however, all those who held these beliefs were workers on ships or in destination markets. A previous survey of Southeast and East Asian students recognized that respondents from a rural residence, and those with more farm animal experience, were less accepting of killing seriously injured or ill animals [25]. Our findings may be related to the high proportion of shipboard and in-market workers who have worked with farm animals that are not from Australia and the relatively high importance and economic value of individual animals to farmers in developing nations [38].

Strong opinions and potential misconceptions exist regarding workers in the live export industry. A survey of the Australian public found they placed higher levels of trust in veterinarians than other stakeholders directly employed in the live export context [16]; however, it was not specifically stated against which other industry stakeholders they were compared. Other reports have highlighted the public’s particular concerns regarding workers involved in transporting animals by sea and those who handle animals in countries receiving Australian livestock [3,13]. Our study countered these sentiments by showing that industry workers’ attitudes toward animal welfare generally did not vary according to their role in the supply chain. The public’s perceptions perhaps reflect systemic industry issues prior to the introduction of the Export Supply Chain Assurance System (ESCAS) in 2011. Despite ESCAS improvements, the media’s framing of events involving poor welfare in destination countries typically aims to promote moral shock rather than providing objective information regarding animal welfare issues within the supply chain [1,4]. Furthermore, incidents occurring outside Australia are rarely put in perspective against production animal welfare issues that exist domestically. Changing negative perceptions of industry workers and viewing them as a competent resource for understanding and implementing welfare regulations will help drive ongoing industry improvement [15].

This study suggests that participants were highly likely to display behavior that led to or optimized welfare outcomes when it was feasible to do so. The ability of stock handlers to deliver good welfare outcomes depends on actual behavioral control and may be hampered by factors beyond the individual’s control [30]. To effect positive interactions, it is necessary to provide people with resources and opportunities to perform the desired behavior and make ongoing improvements to animal welfare [5,22]. Findings suggest that when welfare outcomes do not meet societal expectations, contributing factors are likely to be beyond the control of those directly handling livestock. Industry issues such as availability of resources, inappropriate infrastructure, adverse environmental conditions, lack of training, or a combination of multiple factors [39] are more pertinent causes of undesirable welfare outcomes rather than worker complacency or deliberate mishandling of livestock.

Many people working in the livestock export supply chain outside Australia enter the industry with limited experience handling Australian livestock and develop skills experientially on the job rather than by formal training. Receiving training in effective stock handling practices can significantly improve human–animal interactions [5,15]. The implementation of ESCAS has improved handling and infrastructure in foreign facilities but does apply to domestic facilities or shipboard workers. Although very few participants who did not demonstrate a proficient understanding of animal welfare concepts, the low level of formal training reported identifies this as an area where improvements can be achieved, both domestically and internationally. Industry investment in staff training, maximizing worker retention, and creating a positive workplace environment can significantly improve animal welfare outcomes [15].

Most participants completed the surveys in the workplace at times of high activity (for example, during vessel loading or receival of livestock at a feedlot). However, it is known that emotional valence at the time of decision making can influence behavior [5,30] and survey context does not account for a participant’s affective state, or the influence of external factors [6] when handling livestock. Hence, directly recording observed behavior in the workplace can further improve our understanding of stock handlers’ interactions with animals. Surveying a control group of stock handlers not associated with the livestock export industry, or members of the general public, would add value to this study, as would sourcing information from more pre-export facilities and destination markets. The exclusion of workers from one exporting company reduces the scope of responses to reflect all industry workers accurately.

## 5. Conclusions

Respondents demonstrated a good understanding of animal welfare and displayed positive attitudes and empathy toward working with livestock. People from all sectors of the supply chain showed positive intent toward improving animal outcomes and indicated that taking affirmative action on animal welfare was the social norm in their workplace. Surveyed workers reported a high level of perceived behavioral control around efforts to improve welfare outcomes. These fundamental elements indicate the likelihood of workers displaying behavior that positively influences animal welfare. There were few differences in attitudes, beliefs, or actions between industry roles, indicating that, where feasible, livestock export industry workers will behave in a way that optimizes animal outcomes regardless of their role in the supply chain. Many workers provided descriptions of welfare improvements they had undertaken in the past and suggestions for future improvements. These findings are important for guiding future animal welfare policy and developing communication strategies to deal with the public’s perception of workers within the industry.

## Figures and Tables

**Table 1:** Overall responses to Likert scale questions. Cells are shaded to indicate the fewest (white) to the most frequent (dark green) responses. Right hand columns show the results of ordinal regression analyses. Questions that demonstrated a significant difference in Likert scores by role in industry, and the probability that those responses varied significantly, are highlighted in grey and denoted in bold.

Question	Likert Responses						Hypothesis Test				95% Confidence Interval	
	1	2	3	4	5	n	Wald Chi-Square	df	*p* Value	Odds Ratio	Lower	Upper
**1.** Working with livestock is enjoyable to me	68%	27%	3%	0%	1%	263	3.555	1	0.059	1.117	0.996	1.254
**2.** Livestock do not form relationships with humans	4%	17%	8%	40%	31%	258	0.578	1	0.447	1.037	0.944	1.141
**3.** If I see an animal suffer, I think about it for a long time	38%	36%	12%	11%	3%	257	**8.527**	**1**	**0.003**	**0.867**	**0.788**	**0.954**
**4.** Livestock are stupid	8%	5%	6%	47%	34%	258	**4.212**	**1**	**0.040**	**0.903**	**0.819**	**0.995**
**5.** Livestock are uncontrollable	2%	5%	3%	49%	41%	260	0.223	1	0.637	0.976	0.883	1.079
**6.** Livestock have feelings	56%	33%	6%	3%	2%	261	**9.704**	**1**	**0.002**	**0.851**	**0.769**	**0.942**
**7.** Cattle don’t feel pain	5%	3%	1%	32%	59%	259	0.159	1	0.690	0.979	0.885	1.085
**8.** I can see if livestock are stressed	44%	48%	7%	0%	0%	260	0.235	1	0.628	1.025	0.927	1.134
**9.** Hitting cattle helps when moving them *	1%	2%	30%	37%	31%	259	2.509	1	0.113	1.079	0.982	1.186
**10.** It is acceptable to pull a sheep by a leg to move them *	2%	1%	15%	26%	57%	255	0.000	1	0.998	1.000	0.902	1.108
**11.** Sheep don’t feel pain	1%	1%	2%	35%	61%	259	2.058	1	0.151	1.081	0.972	1.203
**12.** Livestock should be treated with respect	68%	29%	2%	0%	0%	262	0.207	1	0.649	1.026	0.918	1.147
**13.** When moving livestock, it is better to remain calm than shout in a loud voice	56%	33%	4%	4%	3%	262	0.983	1	0.321	0.950	0.859	1.051
**14.** It is important to move livestock slowly	45%	46%	6%	2%	1%	259	0.150	1	0.699	0.980	0.887	1.084
**15.** I am not upset when I see livestock in pain	2%	5%	5%	42%	46%	262	**3.863**	**1**	**0.049**	**1.107**	**1.000**	**1.226**
**16.** It is important for livestock to be able to lie down to rest	60%	34%	4%	2%	0%	260	0.197	1	0.657	0.977	0.882	1.083
**17.** When I see an animal being bullied, I feel sorry for it	52%	40%	6%	1%	2%	261	**14.025**	**1**	**0.000**	**0.822**	**0.741**	**0.911**
**18.** Euthanasia is sometimes necessary in cases of injured or sick livestock	46%	34%	10%	9%	1%	257	3.079	1	0.079	1.092	0.990	1.205
**19.** People in my workplace work hard to care for the livestock	68%	29%	2%	1%	1%	261	1.659	1	0.198	0.930	0.833	1.038
**20.** The welfare of animals is satisfactory in my workplace	58%	38%	3%	1%	1%	261	1.016	1	0.314	0.948	0.856	1.051
**21.** The welfare of livestock in my workplace is important to me	72%	27%	0%	0%	1%	261	0.487	1	0.485	0.961	0.858	1.075
**22.** Sometimes, I cannot do anything to improve poor animal welfare in my workplace	3%	18%	12%	36%	30%	249	1.271	1	0.260	0.944	0.853	1.044
**23.** In the past, I have tried to make improvements to the welfare of animals in my work place	41%	43%	10%	5%	1%	251	0.624	1	0.429	1.042	0.940	1.155
**24.** I feel confident working with livestock *	69%	19%	8%	2%	2%	259	1.022	1	0.312	1.060	0.947	1.187

Likert scale measures: 1 = Strongly agree; 2 = Agree; 3 = Undecided; 4 = Disagree; 5 = Strongly disagree; or, where indicated by * Likert scale measures: 1 = All of the time; 2 = Most of the time; 3 = Sometimes; 4 = Not often; 5 = Never, *n* = number of responses. (Survey questions not in a Likert scale format were omitted from these analyses).

## Data Availability

Data contains personal and industry sensitive information and public sharing of data has not been granted by survey participants. Therefore, limited data is available only upon request due to ethical restrictions.
